# Effect of wearing cotton towel Ihram and plain Ihram on lung function among Hajj Pilgrims

**DOI:** 10.12669/pjms.35.4.727

**Published:** 2019

**Authors:** Sultan Ayoub Meo, Muhammad Iqbal, Adnan Mahmood Usmani, Abdulrahman Abdulaziz Almana, Abdulrahman Hamad Alrashed, Khalid Abdullah Al-Regaiey

**Affiliations:** 1 Sultan Ayoub Meo, Department of Physiology, College of Medicine, King Saud University, Riyadh, Saudi Arabia; 2 Muhammad Iqbal, Department of Physiology, College of Medicine, King Saud University, Riyadh, Saudi Arabia; 3 Adnan Mahmood Usmani, University Diabetes Centre, College of Medicine, King Saud University, Riyadh, Saudi Arabia; 4 Abdulrahman Abdulaziz Almana, Department of Armed Forces Medical Services, King Fahad Armed Forces Hospital, Jeddah, Saudi Arabia; 5 Abdulrahman Hamad Alrashed, Department of Family Medicine, King Faisal Specialist Hospital and Research Centre, Riyadh, Saudi Arabia; 6 Khalid Abdullah Al-Regaiey, Department of Physiology, College of Medicine, King Saud University, Riyadh, Saudi Arabia

**Keywords:** Cotton Ihram, Hajj Pilgrims, Lung Function, Plain Ihram

## Abstract

**Objectives:**

Hajj is the world’s largest gathering to Makkah, Saudi Arabia. Wearing cotton made Ihram is a basic and an essential component of Hajj. The aim of this study was to investigate the lung functions among Hajj pilgrims who were wearing cotton towel ihram (ihram with fibers) compared to those who were wearing plain cotton ihram (ihram without fibers).

**Methods:**

Ninety male, non-smoker, Hajj pilgrim volunteers with age ranged 20-60 years were selected. Forty five of them wore cotton towel ihram and 45 wore plain ihram. A day before leaving for Hajj and wearing ihram (6^th^ Dhu-al-Hijjah) lung function base line parameters of Hajj pilgrims were determined. Hajj Pilgrims continuously wear ihram from 7-10^th^ Dhu-al-Hijjah. In the afternoon of 10^th^ Dhu-al-Hijjah, after removal of ihram, all parameters were repeated and at the completion of Hajj when all pilgrims return to their homes at Riyadh, all parameters were recorded again.

**Results:**

Before wearing Ihram, anthropometric and lung function baseline parameters were recorded, no significant difference was found between the study population. After wearing Ihram on the 7^th^ Dhu-al-Hijjah and its removal on the 10^th^ Dhu-al-Hijjah significant decline in the lung function test parameters was observed among Hajj pilgrims who were wearing cotton towel ihram. Forced Vital Capacity (FVC) 4.30±1.18 vs. 5.03±1.41 (p=0.01); Forced Expiratory Flow 25% (FEF-25%) 4.39±1.94 vs. 5.69±2.84 (p=0.03); Forced Expiratory Flow-50% (FEF-50%) 2.93±1.65 vs. 4.07±2.08 (p=0.01); Forced Expiratory Flow-75% (FEF-75%) 1.02±0.70 vs. 1.66±0.94 (p=0.002) compared to those who were wearing plain ihram.

**Conclusions:**

Lung function test parameters were decreased among the Hajj pilgrims who were wearing cotton towel ihram compared to those who were wearing plain cotton ihram. The pattern of impairment of lung function shows an obstructive peripheral airway lung involvement. It is suggested to conduct further large sample size studies to confirm the present study observations and reach at better conclusions.

## INTRODUCTION

**Fig. 1:**
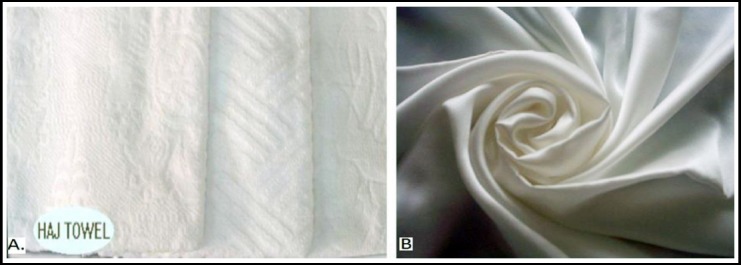
[A] Cotton towel ihram with fibers, [B] Plain cotton ihram without fibers.

Hajj is a unique Islamic ritual where each year about 2.5 million Muslims from all over the world travel to Makkah, Saudi Arabia to perform Hajj during the period 8-12^th^ Dhu-al-Hijjah (Lunar month).^1^ Wearing Ihram is a basic and obligatory component for all male Hajj pilgrims. They continuously wear the ihram since the early morning of 8^th^ Dhu-al-Hijjah and remove it at the afternoon of 10^th^ Dhu-al-Hijjah; and remaining days from 10^th^ to 12^th^ Dhu-al-Hijjah, all Hajj pilgrims perform their remaining Hajj rituals without ihram but must stay in similar environment at Mina (a place at Makkah). Majority of the pilgrims tend to wear cotton towel ihram [Ihram with fibers], which contains cotton fibers (Fig. 1A) which are released into the environment and may become a cause of respiratory complaints. Cotton fibers elicit pro-inflammatory mediators, which are involved in acute or chronic respiratory inflammation.^2^

The respiratory system is the most vulnerable body system for air pollution. Exposure to airborne cotton fiber and associated dust pollutants impairs the various physiological functions, represents a major factor promoting respiratory illness expression, and develops adverse effects on the human health. The respiratory problems are commonly seen during Hajj season.^3^ The hypothesis behind this study is that there are multiple factors, which can impair the respiratory health of Hajj pilgrims. However, we believe that, wearing cotton Ihram with fibers (Fig.1A) is one of the leading factors as it generates and upsurges the respiratory complaints in Hajj pilgrims. The aim of this study was to investigate the lung functions among Hajj pilgrims who were wearing cotton towel ihram (ihram with fibers) compared to those who were wearing plain cotton ihram (ihram without fibers).

## METHODS

Initially we recruited 160 male hajj pilgrims, but after clinical history, we recruited 90 male hajj pilgrims for the research project. For individuals, serial number was allotted for wearing cotton or plain ihram, and adjustments were made in buses for travelling to Makkah, as well as staying in tents at Mina at Makkah city, for research procedure. A day before leaving for a Hajj and wearing ihram on 5^th^ Dhu-al-Hijjah, all the participants were gathered to obtain their base line lung function parameters. On the evening of 6^th^ Dhu-al-Hijjah both groups travelled for Makkah city to perform the Hajj rituals. Forty-five of them dressed up in cotton towel ihram and 45 were in plain ihram. Hajj pilgrims who wore cotton towel ihram were allowed to sit in one bus, and who wore plain ihram were allowed to sit in another bus. On arrival in Makkah city, hajj pilgrims who wore cotton towel ihram were allowed to join one tent for their stay in Mina, a place where all the pilgrims gathered to perform the various rituals of Hajj. Hajj pilgrims who wore plain towel ihram were allowed to join another tent for their stay in Mina and were instructed not to mix-up.

After the removal of Ihram, on the afternoon of 10^th^ Dhu-al-Hajj all the lung function parameters were again obtained. Following completion of all Hajj rituals on the evening of 12^th^ Dhu-al-Hijjah, both groups were advised to sit in the allocated buses to return back to Riyadh. On the 15^th^ Dhu-al-Hijjah, both groups were invited and all the parameters were re-investigated as before.

### Exclusion Criteria

Initially 160 subjects who submitted their applications for hajj were interviewed, and clinical history and physical examinations were conducted. Subjects with any acute or chronic respiratory diseases, anemia, diabetes mellitus, malignancy, and also those who were current or former traditional cigarette smokers, shisha smokers and other tobacco product users were excluded from the study. Subjects who worked in any industry, which generates dust or fumes, were also excluded from the study.^4^,^5^

### Spirometry

Spirometry was conducted using SPIROVIT SP-1 (Schiller, Switzerland) to assess lung function test parameters including “Forced Vital Capacity (FVC), Forced Expiratory Volume in First Second (FEV_1_), Forced Expiratory Ratio (FEV_1_/FVC %), Peak Expiratory Flow (PEF), Forced Expiratory Flow-25% (FEF_25%_); Forced Expiratory Flow-50% (FEF_50%_); and Forced Expiratory Flow-75% (FEF_75%_)”. The lung function test parameters were performed based on the official statement of the American Thoracic Society of Standardization 2005.^6^ Participants were given detailed descriptions of the tests. The tests were performed in the standing position with a nose clip at a fxed time of the day to minimize the diurnal variations. Separate new sterile mouth pieces for each individual were used to prevent any cross infections.

### Ethical Statement

The study was approved by “Institutional Review Board, Ethics Committee, College of Medicine Research Centre, King Saud University, Riyadh, Saudi Arabia (E-14-1245)”.

### Statistical Analysis

The results were analyzed by using the Statistical Package for Social Sciences (SPSS) version 20.0 for Windows. Student’s t-test was applied to test the difference of the means between the two quantitative variables. The level of significance was assumed at p< 0.05.

## RESULTS

The physiological variables, age, height, weight were matched between the Hajj pilgrims who were wearing cotton towel ihram compared to those who were wearing plain cotton ihram (Table-I). The mean age of Hajj pilgrims who were wearing cotton towel ihram group was 36.08±10.94 vs those who were wearing plain cotton ihram 37.22±9.41; height 1.64±0.70 vs 164.0±05; weight 72.69±13.27 vs 71.53±8.94 (Table-I). There was no significant difference in anthropometric variable between the groups (Table-I).

**Table I:** Comparison of anthropometric parameters between Hajj pilgrims wearing cotton towel Ihram compared to Hajj pilgrims wearing plain Ihram (Pre Hajj data).

Parameters	Towel Ihram Group (n=45)	Plain Ihram Group (n=45)	P-Values
Age (years)	36.08 ± 10.94	37.22 ± 9.41	0.630
Height (m)	1.64 ± .07	1.64 ± 0.05	0.706
Weight (kg)	72.69 ± 13.27	71.53 ± 8.94	0.660

Vales are presented in Mean ±SEM

A day before leaving for Hajj and wearing the required Ihram, baseline lung function test parameters were recorded. There was no significant difference in the lung function test parameters among hajj pilgrims wearing cotton towel ihram compared to those who were wearing plain cotton ihram (Table-II). Forced Vital Capacity (4.49±1.53 vs. 4.57±1.28); Forced Expiratory Volume in First Second (2.99±0.61 vs. 3.01±0.63); Forced Expiratory Ratio (71.17±18.11 vs. 67.12±15.67); Peak Expiratory Flow (5.54±2.05 vs. 5.05±2.25); Forced Expiratory Flow-25% (5.10±1.89 vs. 4.82±2.27); Forced Expiratory Flow-50% (3.21±1.59 vs. 3.12±1.94); Forced Expiratory Flow-75% (1.29±0.87 vs. 1.13±0.86) between the Hajj pilgrims who were wearing cotton towel ihram compared to those who were wearing plain cotton ihram (Table-II).

**Table II:** Comparison of lung function parameters between Hajj pilgrims wearing Cotton Towel Ihram compared to Hajj pilgrims wearing Plain Ihram (Pre Hajj data).

Parameters	Cotton Towel Ihram Group (n=45)	Plain Ihram Group (n=45)	P Values
FVC (lit)	4.49 ± 1.53	4.57 ± 1.28	0.827
FEV1 (lit/sec)	2.99 ± .61	3.01 ± 0.63	0.912
FEV//FVC Ratio (%)	71.17 ± 18.11	67.12 ± 15.67	0.305
PEF (lit/ sec)	5.54 ± 2.05	5.05 ± 2.25	0.317
FEF-25% (lit/sec)	5.10 ± 1.89	4.82 ± 2.27	0.549
FEF-50% (lit/sec)	3.21 ± 1.59	3.12 ± 1.94	0.824
FEF-75% (lit/sec)	1.29 ± .875	1.13 ± 0.86	0.430

Vales are presented in Mean ±SEM, NS: Non-Significant

The lung function test parameters were recorded during Hajj on 10^th^ of Dhu-al-Hajj, after the removal of the Ihram. There was a significant decline in the lung function test parameters among Hajj pilgrims who were wearing cotton towel ihrams. Forced Vital Capacity (FVC) 4.30±1.18 vs. 5.03±1.41 (p=0.01); Forced Expiratory Flow-25% (FEF-25%) 4.39±1.94 vs. 5.69±2.84 (p=0.03); Forced Expiratory Flow-50% (FEF 50%) 2.93±1.65 vs. 4.07±2.08 (p=0.01); Forced Expiratory Flow-75% (FEF-75%) 1.02±0.70 vs. 1.66±0.94 (p=0.002) compared to those who were wearing plain ihram (Table-III). However, there was no significant difference in Forced Expiratory Volume in First Second (FEV1) 2.89±0.75 vs. 3.56±1.09 (p=0.386); Forced Expiratory Ratio (FEV1/FVC Ratio) 68.59±13.12 vs. 71.14±13.08 (p=0.543) and Peak Expiratory Flow (PEF) 5.15±1.9 vs. 6.07±2.74 (p=0.127) between the Hajj pilgrims who were wearing cotton towel ihram compared to those who were wearing plain cotton ihram (Table-III).

**Table III:** Comparison of lung function parameters between Hajj pilgrims wearing Cotton Towel Ihram compared to Hajj pilgrims wearing Plain Ihram (During Hajj data).

Parameters	Towel Ihram Group (n=45)	Plain Ihram Group (n=45)	P Values
FVC (lit)	4.30 ± 1.18	5.03 ± 1.41	0.018
FEV1 (lit/sec)	2.89 ± .755	3.56 ± 1.09	0.386
FEV//FVC Ratio (%)	68.59 ± 13.12	71.14 ± 13.08	0.543
PEF (lit/ sec)	5.15 ± 1.99	6.07 ± 2.74	0.127
FEF-25% (lit/sec)	4.39 ± 1.94	5.69 ± 2.84	0.031
FEF-50% (lit/sec)	2.93 ± 1.65	4.07 ± 2.08	0.013
FEF-75% (lit/sec)	1.02 ± .70	1.66 ± 0.94	0.002

Vales are presented in Mean ±SEM; NS= Non-Significant

The lung function test parameters were also recorded after Hajj, and a significant decline was found among Hajj pilgrims who were wearing cotton towel ihrams in Peak Expiratory Flow (PEF) 5.69±1.87 vs. 6.78±2.08 (p=0.015); Forced Expiratory Flow 25% (FEF-25%) 4.97±1.97 vs. 6.30±2.08 (p=0.005) compared to those who were wearing plain cotton ihram (Table-IV).

**Table IV:** Comparison of lung function parameters between Hajj pilgrims wearing Cotton Towel Ihram compared to Hajj pilgrims wearing Plain Ihram (After Hajj data).

Parameters	Towel Ihram Group (n=45)	Plain Ihram Group (n=45)	P-Values
FVC (lit)	4.32 ± .95	4.56 ± 1.01	0.314
FEV1 (lit/sec)	2.80 ± .90	3.03 ± 0.93	0.060
FEV//FVC Ratio (%)	70.45 ± 16.00	72.31 ± 14.11	0.465
PEF (lit/ sec)	5.69 ± 1.87	6.78 ± 2.08	0.015
FEF-25% (lit/sec)	4.97 ± 1.97	6.30 ±2.08	0.005
FEF-50% (lit/sec)	3.01 ± 1.42	3.55 ± 1.85	0.134
FEF-75% (lit/sec)	1.18 ± .85	31.19 ± 175.60	0.301

Vales are presented in Mean ±SEM; NS= Non-Significant

## DISCUSSION

This is the first novel study which presents an assessment of lung function parameters among Hajj pilgrims. A significant decline in the lung function test parameters was found among Hajj pilgrims who were wearing cotton towel ihrams compared to those who were wearing plain ihrams. These parameters show some evidence that after Hajj, the lung function parameters of pilgrims wearing cotton towel ihrams decreased and this decline shows a peripheral obstructive airway lung impairment. However, these parameters were reversed a few days after the removal of the Ihram.

Hajj is one of the world’s largest annual mass gathering on the planet. Approximately 2.5 million people from all over the world assemble annually at Makkah, Saudi Arabia to perform Hajj.^7^ Shakir et al.^8^ found that during Hajj, the most frequent complaints were related to respiratory problems. There are many factors promoting the respiratory diseases which include dust, air pollution^9^ and viral infections.^10^ Wearing cotton towel ihram with fibers increases the chances of releasing cotton fibers into the environment and may become a cause of respiratory complaints.

Environmental pollution enhances the global burden of allergic and respiratory diseases, including chronic obstructive pulmonary disease (COPD), asthma, pneumonia, and other respiratory problems. The links between environmental pollution and respiratory disease are multifaceted and current studies have showed an increased significance of air pollution associated respiratory problems.^11^ Exposure to environmental pollutants leads to respiratory diseases and other long-lasting health problems.^12^ A quarter of the diseases faced by mankind today occur due to exposure to dust, fumes and fibers in the environment.

Dangi and Bhise^13^ conducted a study among age and gender-matched male cotton mill workers working in weaving and spinning areas. These cotton mill workers showed a significant decrease in “Forced Expiratory Volume in First Second, Forced Expiratory Ratio (FEV1 / FVC Ratio) and Peak Expiratory Flow”.

Paudyal et al.^14^ conducted a study among subjects working in the garments, carpets and weaving cotton industry. The authors identified that exposure to inhalable cotton dust was associated with significant reduction in “Forced Vital Capacity (FVC) and Forced Expiratory Volume in First Second (FEV1)”. Similarly, Kahraman et al.^15^ found that cotton dust exposure effected the lungs and reduces the FEV1 and FEV1/FVC ratio in cotton mill workers. These findings support the present study findings that exposure to cotton dust in any form decreases the pulmonary function.

Beshir et al.^16^ reported that exposure to cotton fibers and its associated dust in environments causes changes in the lung functions. A noticeable reduction was identified in Forced Vital Capacity and Forced Expiratory Volume in First Second” in the exposed group compared to the control. The ventilatory functions were positively correlated with the duration of exposure.^16^

Jing et al.^17^ reported that prolonged exposure to cotton fibers and its dust is allied with respiratory complaints and decline in FEV1. Moreover, it has also been demonstrated that subjects exposed to cotton fibers and its associated dust had larger and more frequent declines in FEV1 compared to silk exposed subjects.^18^ Similarly, the present study findings show that lung function test parameters, “Forced Vital Capacity; Forced Expiratory Flow-25%; Forced Expiratory Flow-50%; Forced Expiratory Flow-75%” among Hajj pilgrims who were wearing cotton towel ihrams were decreased compared to those who were wearing plain ihram.

### Limitations of study

The study has some limitations, which relates to the small sample size. Hajj is the world’s largest annual gathering where approximately 2·5 million pilgrims arrive from all over the world to perform Hajj, therefore, conducting research studies in such a mass gathering is not an easy task. The investigator led both groups separately from Riyadh to Makkah, Saudi Arabia. However, during Hajj at some point, both groups were sitting collectively and it was difficult to separate them. Moreover, in addition to cotton towel Ihram fibers, there were some confounding factors such as dust, mass gathering, close contact and viral infections during the Hajj period which may cause respiratory problems including lung function impairment.

## CONCLUSIONS

Lung function parameters among Hajj pilgrims who were wearing cotton towel ihrams were decreased compared to those who were wearing plain ihram. The pattern of impairment of lung function shows obstructive peripheral airway lung involvement. However, one week after the removal of the Ihram, the lung function test parameters were reversed. It is advised to conduct further large sample size studies to confirm the present study observations to reach at better conclusions.
